# Development of a β-Lactoglobulin Sensor Based on SPR for Milk Allergens Detection

**DOI:** 10.3390/bios8020032

**Published:** 2018-03-27

**Authors:** Jon Ashley, Roberta D’Aurelio, Monika Piekarska, Jeff Temblay, Mike Pleasants, Linda Trinh, Thomas L. Rodgers, Ibtisam E. Tothill

**Affiliations:** 1 Advanced Diagnostics and Sensors Group, Cranfield University, Cranfield MK43 0AL, UK; r.daurelio@cranfield.ac.uk (R.D.); monika.piekarska9@gmail.com (M.P.); 2 Department of Micro- and Nanotechnology, Denmark Technical University, 2800 Lyngby, Denmark; 3 Safety and Environmental Assurance Centre, Colworth Science Park, Unilever plc, Sharnbrook, Bedford MK44 1LQ, UK; jeff.temblay@scienceexchange.com (J.T.); Mike.Pleasants@unilever.com (M.P.); 4 School of Chemical Engineering and Analytical Science, University of Manchester, Oxford Road, Manchester M13 9PL, UK; linda.trinh@manchester.ac.uk (L.T.); Tom.Rodgers@manchester.ac.uk (T.L.R.)

**Keywords:** allergen, milk protein, β-lactoglobulin (BLG), surface plasmon resonance (SPR), biosensor

## Abstract

A sensitive and label-free surface plasmon resonance (SPR) based sensor was developed in this work for the detection of milk allergens. β-lactoglobulin (BLG) protein was used as the biomarker for cow milk detection. This is to be used directly in final rinse samples of cleaning in-place (CIP) systems of food manufacturers. The affinity assay was optimised and characterised before a standard curve was performed in pure buffer conditions, giving a detection limit of 0.164 µg mL^−1^ as a direct binding assay. The detection limit can be further enhanced through the use of a sandwich assay and amplification with nanomaterials. However, this was not required here, as the detection limit achieved exceeded the required allergen detection levels of 2 µg mL^−1^ for β-lactoglobulin. The binding affinities of the polyclonal antibody for BLG, expressed by the dissociation constant (K_D_), were equal to 2.59 × 10^−9^ M. The developed SPR-based sensor offers several advantages in terms of label-free detection, real-time measurements, potential on-line system and superior sensitivity when compared to ELISA-based techniques. The method is novel for this application and could be applied to wider food allergen risk management decision(s) in food manufacturing.

## 1. Introduction

Milk allergies have been a major concern for public health, especially in children [1]. A food allergic reaction is defined as an immune reaction to a constituent in a food product such as a protein through the production of immunoglobulin E (IgE) [2]. The body produces histamine and other chemicals such as cytokines, which may result in several symptoms such as inflammation and in severe reactions, fatal anaphylaxis. A key concern for food manufacturers is the detection of food allergen cross-contamination within wider food manufacturing processes, which involves resource-intensive steps such as swabbing food processing plants and routine testing of final products. It is known that relatively low levels of milk allergens can cause reactions in allergic individuals: this is indicated by the Voluntary Incidental Trace Allergen Labelling (VITAL) framework. In particular, the VITAL framework proposes that a reference dose of at least 0.1 mg of milk protein per consumer portion of product is a sufficient basis for food manufacturers to apply precautionary allergen labelling. Currently, there is a desire to develop cheap reliable on-line or at-line tests for detecting milk protein allergens in a variety of different food matrices [3] or within food manufacturing plants. Rocket immune electrophoresis is a classic technique, which is used in the detection of casein protein although the technique is largely obsolete [4]. 

Immunological-based tests such as lateral flow-based assays (LFAs) and enzyme-linked immune sorbent assay (ELISA) are the most widely used methods for the detection of milk allergens with several commercial kits being offered on the market. LFAs are a quick and simple method for detecting milk allergens [5] and they have proven to be cost effective, but the possibility for quantitative analysis using this type of assay is currently limited. ELISA-based assays, which use antibodies in either direct or sandwich-based assays, have been demonstrated previously in the detection of a number of milk allergens [6,7,8,9]. Recent studies have suggested large variations in milk allergen recoveries measured using a number of these commercial kits, which is problematic where accurate determination of allergens is required [10]. LC-MS has proven to be the benchmark technique for the sensitive quantitative analysis of milk allergens in a wide range of food matrices [11,12]. The technique offers good sensitivity and precision but the lengthy sample preparation and expensive instrumentation means that analysis needs to be carried out in a dedicated lab. Therefore, a more rapid and efficient detection method for traces of milk allergens in food production facilities is needed and if they are adapted for on-line analysis, then that will be highly beneficial for food manufacturers. 

Biosensors are an attractive alternative to traditional techniques and offer comparable sensitivities and selectivity while allowing for on-line and real-time detection [13,14,15]. Several studies have demonstrated the use of biosensors in the detection of milk proteins [16,17,18,19,20]. Ito et al. demonstrated the analysis of β-lactoglobulin using a flow-based QCM sensor with a detection limit down to 1 ppm [21]. Billakanti et al. [22], described a surface plasmon resonance (SPR) biosensor (ProteOn XPR36, Bio-Rad) for the simultaneous detection of five milk proteins in dairy products reporting different detection ranges. Indyk and Filonzi [23] and Muller-Renaud et al. [24] used SPR biosensors for lactoferrin and α-s1-casein in a variety of milk products and gave a Limit of Detection (LOD) of 19.9 mg mL^−1^ and 0.87 mg mL^−1^, respectively. Recently, an SPR sensor was developed in our previous work for the detection of α-casein protein as an allergen detection in wash samples from cleaning in-place systems (CIP) of the food manufacturing process, achieving an LOD of 58 ng mL^−1^ and adequate sample recoveries [25]. However, whey proteins cannot be detected using a casein-based sensor. Therefore, the development of a β-lactoglobulin sensor is important for whey proteins allergen detection. 

In the current work, an immunoassay-based SPR sensor was developed and optimised for the detection of β-lactoglobulin for future application in cleaning in place (CIP) final-rinse water samples. SPR sensors offer distinct advantages for this application in that they are fully automated, versatile, flexible, and provide rapid and real-time analysis. The sensors also have the potential to provide on-line or at-line analysis for multiple samples. β-lactoglobulin is a significant fraction of milk protein and the detection of β-lactoglobulin would be a useful marker for monitoring levels of milk as well as milk whey fraction during the CIP process in combination with other known allergen sampling methods such as surface swabbing. The developed sensor showed sub ppm sensitivity, good selectivity and was able to detect β-lactoglobulin levels. The sensor can be combined with the casein sensor for the complete detection of milk and whey fraction allergens when these are used separately in food manufacturing processes.

## 2. Materials and Methods

### 2.1. Materials and Equipment

Phosphate-buffered saline (PBS), β-lactoglobulin (BLG) from bovine milk, bovine serum albumin (BSA), sodium acetate, 11-mercaptoundecanoic acid 95% (MUDA), and sulphuric acid 95.0–98.0% were purchased from Sigma Aldrich (Saint Louis, MO, USA). Sodium chloride (NaCl), sodium hydroxide (NaOH), 2-(*N*-morpholino)ethanesulfonic acid (MES), HEPES (4-(2-hydroxyethyl)-1-piperazineethanesulfonic acid), HBS-EP = 10 mM Hepes, 150 mM NaCl, 3.4 mM EDTA, 0.005% Tween-20) were supplied by Fischer Scientific UK (Loughborough, UK). *N*-hydroxysuccinimide (NHS) and 1-ethyl-3-(3-dimethylaminopropyl) carbodiimide (EDC) were purchased from Thermo Scientific (Waltham, MA, USA). Ethanolamine and ethanol were bought from Fluka analytical (Buchs, Switzerland). Hydrogen peroxide 35% was supplied by Acros Organics (Geel, Belgium) and ethylenediaminetetraacetic acid (EDTA) by BOH. Ultrapure water was obtained from a Milli-Q-water system. Oxygen-free nitrogen was bought from BOC (Manchester, UK). Sheep anti-bovine β-lactoglobulin B polyclonal antibody (BLG Ab) was purchased from AbD Serotec (Kidlington, UK). Mouse IgG (mouse IgG) was supplied by Abcam (Cambridge, UK). All obtained chemicals were of analytical grade and were used without further purification.

All the SPR experiments were performed using the SPR 2/4 instrument and SPR affinity sensor chips obtained from Sierra Sensors (Hamburg, Germany). 

### 2.2. Preparation of the Sensor Surface

Bare sensor chips were cleaned by treating with piranha solution (3:1 H_2_SO_4_ and H_2_O_2_). A self-assembly monolayer (SAM) was then formed by submerging the gold chip in a degassed solution of 5 mM solution of 11-mercaptoundecanoic acid 95% (MUDA), in ethanol (50 mL) for at least 24 h. Subsequently, the chips were removed from the solution and rinsed with water and ethanol before drying in nitrogen. The dry chips were then used to develop the immunoassay on the sensor surface and stored at 4 °C until use. A dry chip was docked into the SPR instrument ready for use. The flow rate of the SPR was set at 25 µL min^−1^ with 10 mM PBS pH 7.4, and the temperature kept at 25 °C. The sensor surface was activated by injecting a mixture of 0.1 M NHS/0.4 M EDC for 2 min. The polyclonal BLG antibody was then injected over spot 1 for 4 min followed by injection of the control antibody (mouse IgG) on spot 2 using the optimised pH and concentration of the antibodies. Spots 1 and 2 were blocked by injecting 50 µg mL^−1^ of BSA onto both spots for 4 min followed by a 4 min injection of 1 M Ethanolamine. 

### 2.3. Optimisation of Immobilisation Conditions

Optimisation studies were carried out to find the best pH for antibody immobilisation as well as to establish the optimal antibodies concentration (from 50 µg mL^−1^ up to 500 µg mL^−1^). In order to establish the ideal pH value, sodium-acetate buffer at the following pH values: 4.0, 4.5, and 5.0 was used. PBS buffer at pH 7.4 was also tested to examine the effect of higher pH on the antibody immobilisation. Buffers with pHs over 7.4 were not tested due to their detrimental effect on antibody immobilisation performance due to a decrease in electrostatic interactions between the sensor surface and the antibody. The anti-BLG Ab was suspended in each pH-adjusted buffer, while its concentration was kept constant. The different anti-BLG Ab suspensions were injected onto the activated sensor surface and the signal was recorded to establish the highest readings. The SPR response (expressed in resonance unit, RU) was evaluated to select the best pH condition. 

Following this, the optimal pH condition was used to establish the best antibody concentration. Specifically, several anti-BLG Ab concentrations (50 µg mL^−1^, 75 µg mL^−1^, 100 µg mL^−1^, 150 µg mL^−1^, 500 µg mL^−1^) were tested for their immobilisation efficiency and the one that yielded the highest SPR response (RU) was selected for further testing within this study. 

### 2.4. BLG Cumulative Binding Assay Optimisation

For the cumulative binding assay, anti-BLG Ab were immobilised on spot 1 (active) according to the optimised protocol. Mouse IgG at a concentration of 70 µg mL^−1^ was immobilised on spot 2 and used as a control. In all the experiments, the active and control biosensor-array surfaces (Spots 1 and 2, respectively) were blocked by injecting 50 µg mL^−1^ of BSA onto both spots for 4 min, followed by a 4 min injection of 1 M ethanolamine.

An optimisation study was carried out to reveal the best pH condition and buffer composition to carry out the cumulative binding assays. Specifically, several buffer compositions with different pH values (from 4.0 to 7.4) with or without additive concentrations were explored, as summarised in Table 1. 

**Scheme 1:**
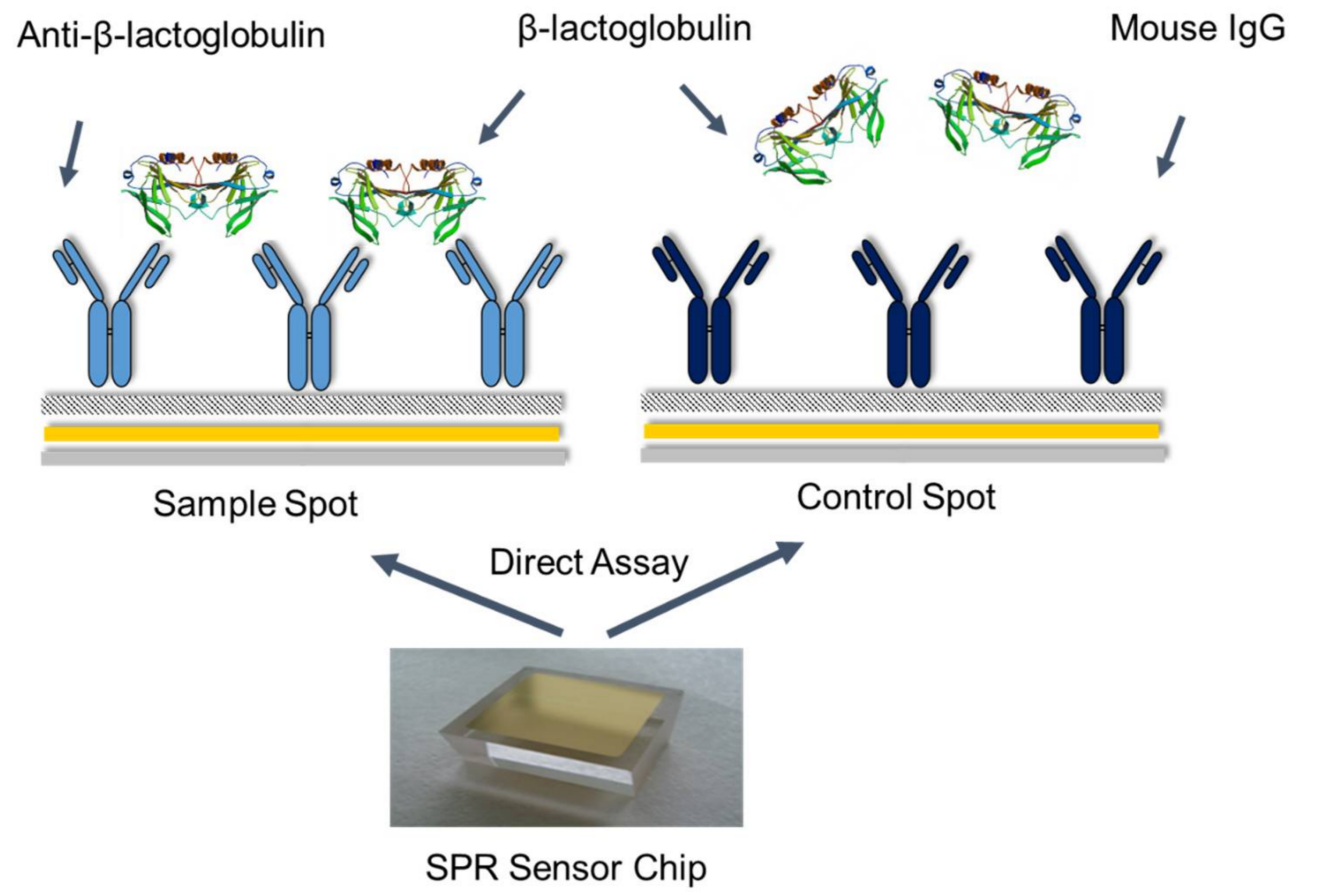
Schematic representation of the surface plasmon resonance (SPR) direct assay for β-lactoglobulin (BLG) detection.

The response was taken just after the end of the injection and normalised by subtracting the blank and the control response from spot 2 readings. All these studies were performed by setting the flow rate to 25 µL min^−1^ with sequential injections of BLG standards (0.488–1000 µg mL^−1^) for 4 min over spots 1 and 2. The data was processed by an SPR-2 Analyser v 3.1.10.0 (Sierra Sensors, Hamburg, Germany) and statistically analysed. The BLG calibration curve was plotted. The limit of detection (LOD) was measured by calculating three × standard deviations (s.d.) of the blank signal and extrapolating the response in the calibration plot to achieve the concentration. All injections were carried out in triplicate. Scheme 1 shows the schematic representation of the SPR direct assay principle for BLG detection. 

## 3. Results and Discussion

### 3.1. Sensor Chip Preparation

The immobilisation of the antibodies was conducted by first forming the self-assembled monolayer on the gold sensor chip. The gold chip was submerged in a degassed solution of 11-mecaptodecanoic acid dissolved in ethanol for at least 24 h, as the presence of oxygen can be detrimental to the formation of the SAM layer. The optimised conditions required for producing the highest degree of immobilisation was studied at different pH values (4.0, 4.5, 5.0, 7.4) using sodium-acetate buffer and PBS with the same antibody concentration. These pH values were used to maximise the degree of immobilisation of antibodies and to encourage electrostatic interactions between the activated ester group on the surface of the sensor and the antibodies. As the isoelectric point (pI) values of antibodies are around 7–9, pH values above 7.4 were not tested as the expected net negative charge of the antibody would act to impede the immobilisation process in this case. 

**Figure 1:**
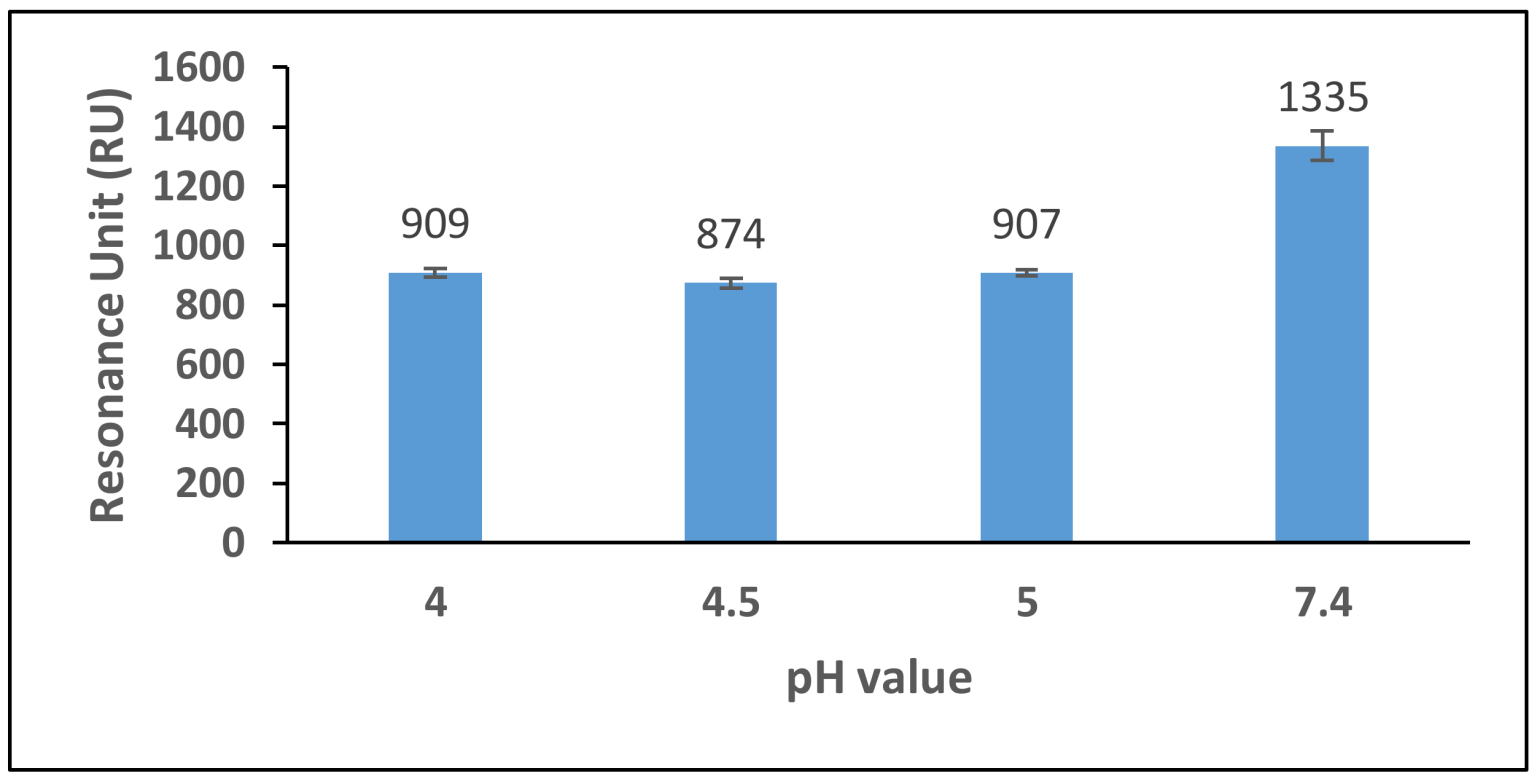
Comparison of anti-BLG polyclonal antibody (150 µg mL^−1^) binding to the sensor chip surface at different sodium-acetate buffer pH values (4.0, 4.5 and 5.0) and PBS at pH 7.4. The running buffer used was 10 mM PBS, pH 7.4.

The pH scouting studies revealed that the best immobilisation of antibodies occurred when anti-BLG Ab was suspended in PBS buffer pH 7.4, reaching a value as high as 1335 RU (Figure 1). Consequently, this buffer was used to carry out the concentration scouting assays, which aimed to determine the optimum antibody concentration required for the sensor surface functionalisation. 

**Figure 2:**
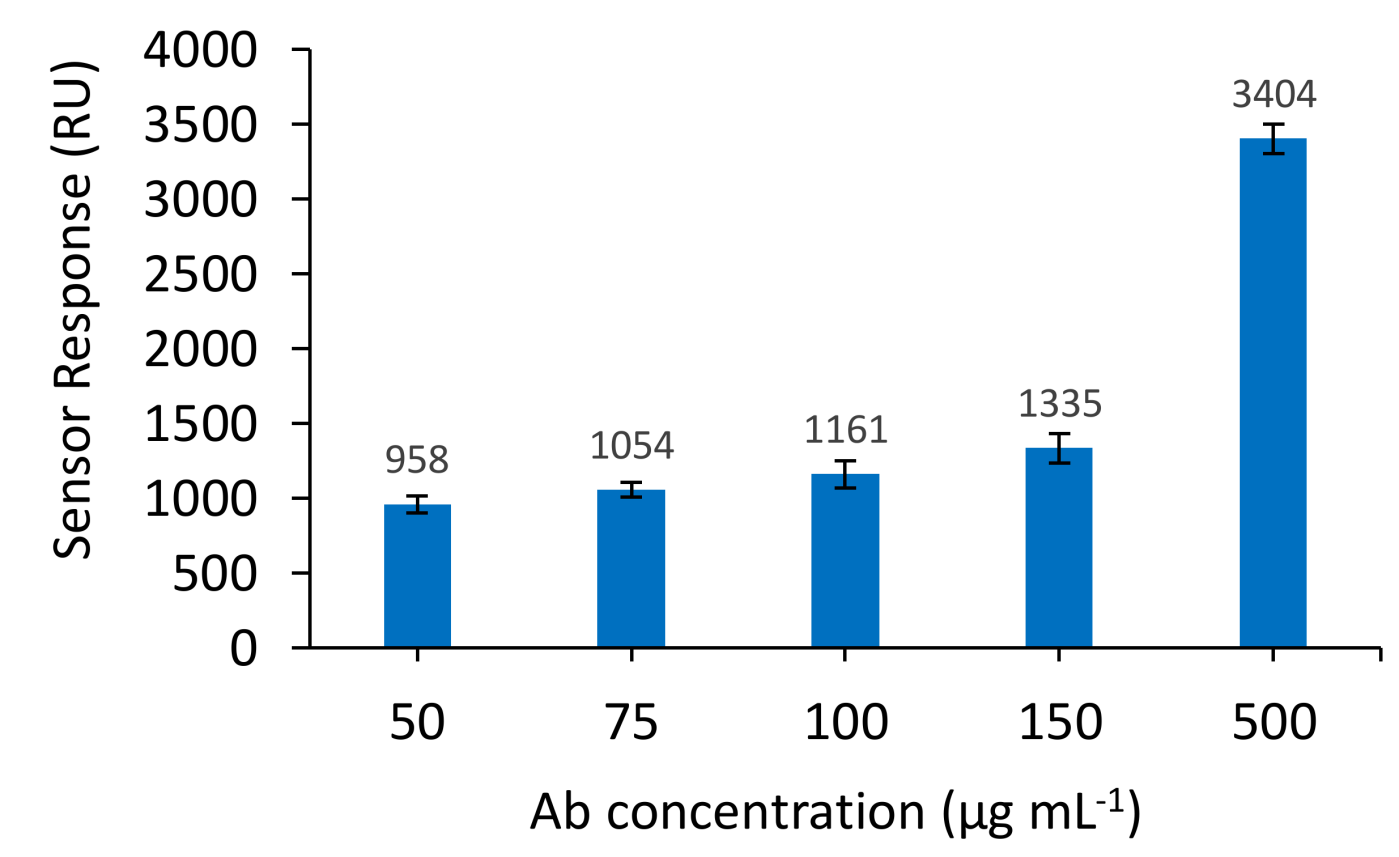
Comparison of different anti-BLG polyclonal antibody concentrations binding to the sensor chip surface. Antibody concentrations were prepared in PBS buffer pH 7.4. The running buffer used was 10 mM PBS. Results are the average of two sensors.

The concentration resulting in the most efficient immobilisation process was then selected as optimal. Specifically, several anti-BLG antibody concentrations were immobilised onto different SPR sensor spots (50 µg mL^−1^, 75 µg mL^−1^, 100 µg mL^−1^, 150 µg mL^−1^, 500 µg mL^−1^) and the sensor response was recorded. A concentration of 500 µg mL^−1^ gave the highest response (3404 RU) and this concentration was used for all subsequent experiments (Figure 2) in order to ensure high sensitivity of the direct assay format.

A concentration above 500 µg mL^−1^ was not explored, as this concentration was considered to be the highest possible concentration in terms of cost/benefit ratio of the sensor development.

### 3.2. β-lactoglobulin Binding Assay

**Figure 3:**
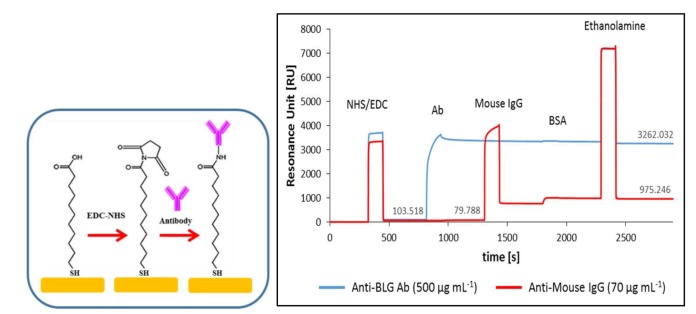
Real-time sensorgram of the anti-BLG polyclonal antibody, mouse IgG immobilisation on the sensor surface and blocking of the unreacted surface with BSA and ethanolamine. The running buffer used was 10 mM PBS, pH 7.4.

All cumulative assays were carried out by immobilising anti-BLG Ab on spot 1 and mouse IgG Ab on spot 2. A typical SPR sensorgram is shown in Figure 3. The carboxylic group on the SAM was activated by injecting a mixture of NHS and EDC, giving a response change of 100 RU. This was followed by the injection of the anti-BLG antibody and mouse IgG control antibody sequentially. The sensor spots were then blocked by injection of BSA followed by ethanolamine, giving final responses of 3262 RU and 975 RU for the active and control spots, respectively. The differences in responses of the two antibodies may be due to the differences in origin of the host species. 

**Figure 4:**
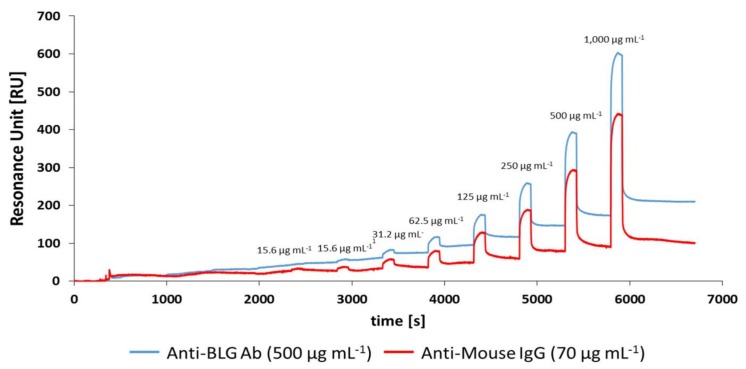
Real-time sensorgram of BLG cumulative assay (0.49–1000 µg mL^−1^) using MES, pH 5.5, as the running buffer.

To establish the best running buffer, in terms of composition and pH, an optimisation study was set up. When PBS, pH 7.4; HEPES, pH 6.8 or HBS-EP (10 mM HEPES, 150 mM NaCl, 3.4 mM EDTA, 0.005% Tween^®^ 20), pH 7.4 were used as a running buffer, no significant binding of the analyte to the anti-BLG Ab could be observed (data not shown). Since BLG is more stable at lower pH values [26], the next tested buffer was MES, pH 5.5. In this case, a significant binding between BLG and the immobilised antibody on the SPR sensor chip could be observed when compared to the control spot array (Figure 4). The sensor displayed a greater response upon injection of BLG towards anti-BLG when compared to the anti-mouse IgG, allowing for a large specific response.

The following buffers with even lower pH were also examined to enhance binding: 0.1 M sodium acetate, pH 4.0, and 0.1 M Glycine-HCl, pH 3.0. Nevertheless, a decrease of binding was observed. Buffers with pH values over 8 were not investigated since BLG conformational changes occurring at a pH range from 8 to 10 have been proven to decrease the immuno-reactivity of the BLG, due to the neutralisation of Lys in position 69. This residue has been considered crucial for the bond between the monoclonal antibody and BLG [27]. To decrease the signal on the control spot, two additives (NaCl and Tween^®^ 20) were tested. Even in this case, no improvement has been achieved (data not shown). Therefore, it can be concluded that MES 10 mM, pH 5.5 was the ideal running buffer.

**Figure 5:**
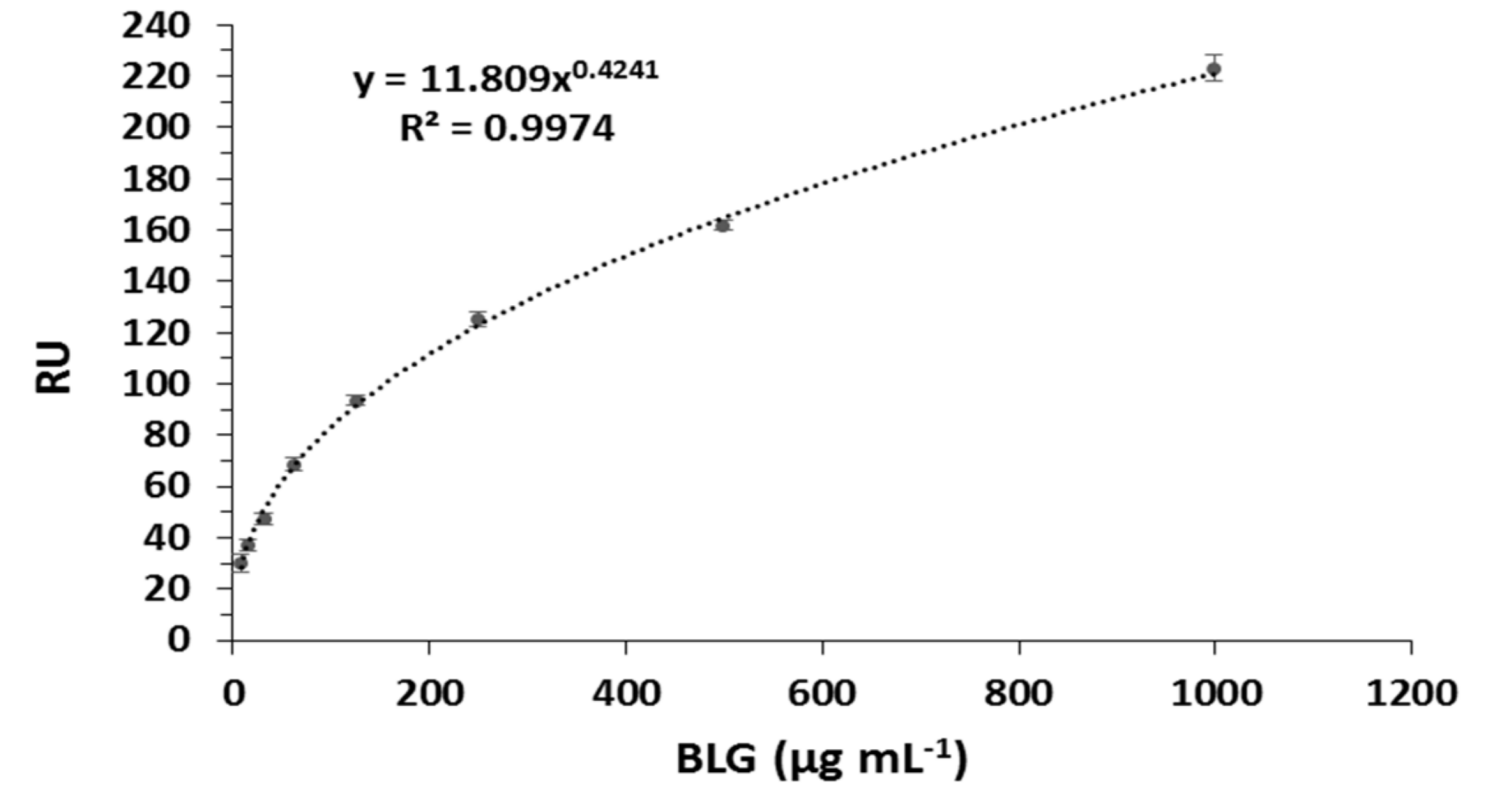
Non-linear calibration curve for the BLG SPR immunosensor, R^2^ = 0.9974.

Once optimised, a cumulative binding assay was performed in triplicate by injecting BLG protein (0.49–1000 µg mL^−1^) and measuring the response. The relative response was normalised by subtracting the response of BLG towards IgG antibody from the response of the BLG towards the anti-BLG antibody. Non-linear regression curves of the relative responses of the BLG sensor against BLG concentration were constructed and are shown in Figure 5. 

The calibration curve shows good non-linear correlation and as the concentration of BLG increases, the sensor response starts to reach saturation. The limit of detection of the SPR biosensor was determined from the relative standard deviation of three blank injections. The LOD was determined and was found to be as low as 0.164 μg mL^−1^. The assay sensitivity can be further improved by using sandwich assay format and amplified with gold nanoparticles. The kinetic fitting study was performed by the Langmuir 1:1 binding model and showed a K_D_ equal to 2.59 × 10^−9^ M, thus providing evidence of affinity between the polyclonal antibody and the BLG analyte. Therefore, the results suggest that the proposed sensor can be used in the detection of BLG in the food production process to detect cross-contamination events.

In comparison to most of the methods reported in the current literature (Table 2), the developed sensor platform in this work aims at detecting traces of BLG in final rinse water samples rather than in food samples.

Therefore, the preferred characteristics are the rapid assay response and an LOD level which is below the required allergen detection levels of 2 ppm [24,25]. Although a direct comparison cannot be made, the developed immuno-based SPR sensor is generally faster and also less expensive and laborious than Mass Spectrometry (MS) or reverse-phase high performance liquid chromatography (RP-HPLC). Compared to the SPR immune-based platform for milk protein detection reported by Billakanti et al. [22], our SPR immunosensor uses a different automated SPR instrument, which is cost effective and includes a blank (reference) as well as a negative control (mouse IgG), thus making the results reliable for this application. Furthermore, our immunosensor was based on direct detection of BLG, whereas Wu et al. [31] used a sandwich assay format. Notably, the developed SPR immunoassay, developed in a direct format, can provide the results in real time, thus being faster and easier to perform than the commercially available immunobased kit (Table 3). 

Immuno-based lateral flow devices can provide only qualitative results, while ELISA (Enzyme Linked Immunosorbent Assay) kits require time-consuming procedures and skilled technicians. Furthermore, as SPR works under microfluidic condition. With further development and validation studies, the biosensor can be integrated within CIP processes, thus allowing for a continuous monitoring of milk allergen residues after the cleaning procedures. 

Milk has ~26 allergens of which caseins represent the casein fraction (80%) and whey fraction (20%) of milk containing BLG. Many of the commercially available kits (ELISA) are also targeted to detect ranges of different proteins or different structures of a single protein or used to quantify total protein content, e.g., “total casein”, “total whey” and “total milk” detection kits (https://www.ncbi.nlm.nih.gov/pubmed/20735137). Allergen detection kits are also specifically modified to these same allergens during food processing–partial hydrolysis, heat treatment, denaturation/aggregation (https://www.ncbi.nlm.nih.gov/pubmed/19641908). As our work currently only looks at the detection of naturally-occurring BLG, the issue of what range of target analytes should be targeted for industrial use in commercially available kits will also apply to biosensor development. Hence, further work is needed to investigate the above points for the final sensor design for BLG analysis. Sample handling procedures and sensor surface blocking optimisation also require thorough investigation before this sensor can be applied for real CIP samples analysis. 

## 4. Conclusions

In this paper, we successfully demonstrated the use of an SPR biosensor in the detection of β-lactoglobulin. The main parameters, such as antibody concentration, pH and the contents of the running buffer were optimised, thus providing an insight into further BLG biosensors development. The optimised SPR-based sensor was successfully fabricated and tested, showing good sensitivity, with an LOD of 0.164 µg mL^−1^. Compared to conventional methods currently in use, this SPR biosensor offers a real-time method based on a direct assay format and performed in an automated microfluidic system, thus reducing time and human labor cost. Due to these advantages, the developed BLG-SPR biosensor can be considered a valuable analytical tool for the monitoring of BLG cross-contamination events in different manufacturing processes required for food, medicine and cosmetics. Thus, the milk allergen SPR biosensor could be used as a tool to support milk allergen safety risk management by industry. 

Further work will look at utilising the same technology to generate antibody-based SPR to detect other sources of food allergens that are of interest to food manufacturing processes, e.g., peanut, egg, etc. It would also be of interest to test the sensor’s ability to detect the absence of residual protein in a CIP validation study, as conducted by [32]. It is also clear from the literature that milk protein standards need to be standardised.

## Figures and Tables

**Table 1:** Buffers investigated for the cumulative assay optimisation study.

Buffer	Concentration (mM)	pH	Additives
Sodium acetate	100 mM	4	-
Glycine-HCl	100 mM	3.0	-
PBS	10 mM	7.4	-
HEPES	10 mM	6.8	-
HBS-EP	10 mM	7.4	150 mM NaCl 3.4 mM EDTA, 0.005% Tween^®^ 20
MES	10 mM	5.5	-
MES	10 mM	5.5	NaCl (50 mM, 100 mM, 150 mM)
MES	10 mM	5.5	0.05% Tween^®^ 20

PBS = Phosphate-buffered saline; HEPES = (4-(2-hydroxyethyl)-1-piperazineethanesulfonic acid); HBS-EP = 10 mM Hepes, 150 mM NaCl, 3.4 mM EDTA, 0.005% Tween-20); MES = 2-(*N*-Morpholino) ethanesulfonic acid.

**Table 2:** A list of the different methods reported for the detection of milk allergens and their limit of detection (LOD).

Allergens	Test Type	Matrix	LOD	Reference
BLG	MS	Fruit juice	1 μg mL^−1^	[28]
BLG	IC dipstick	Cookie, Dumplings fried/steamed, Jelly, Pickles in Vinegar/soy, Potato salad, Sauce, chicken hot dog	5 μg g^−1^	[29]
BLG	RP HPLC	Whey proteins	12 μg mL^−1^	[30]
Whey proteins	SPR	Milk and other milk products	-	[22]
BLG	SPR	-	5.54 ng mL^−1^	[31]
CASs and BLG	IC-dipstick	Chicken meatball or burger, cookie, Dumplings fried/steamed, sauce, jelly, Potato salad, Pickles in Vinegar/soy	5 µg g^−1^	[29]
Whey proteins (ALA, BLG)	Mass Spectrometry	Fruit juice	1 µg mL^−1^	[28]

MS = Mass Spectrometry; IC dipstick = Immunochromatographic dipstick; RP HPLC = Reversed-phase high performance liquid chromatography; SPR = Surface plasmon resonance.

**Table 3:** List of commercially available assay kits for milk allergens detection.

KIT Name	Test Format	Target	LOD	Time Required
Reveal for Total Milk Allergen (Neogen)	LFD	Casein and whey	5 g mL^−1^	5 min
Beta Lactoglobulin Residue ELISA (Oxoid)	ELISA Sandwich, quantitative	β-lactoglobulin	0.1 µg mL^−1^	45 min
Alert for Total Milk Allergen (Neogen)	ELISA Sandwich, quantitative	Milk proteins	5 g mL^−1^	30 min
Veratox for Total Milk Allergen (Neogen)	ELISA Sandwich, quantitative	Milk proteins	5 g mL^−1^	30 min
Beta-lactoglobulin ELISA Kit (Crystal Chem)	ELISA Sandwich, quantitative	β-lactoglobulin in food	0.3 µg g^−1^	-
AgraQuant^®^ ELISA ß-Lactoglobulin (Romer Labs)	ELISA Sandwich, quantitative	β-lactoglobulin in food	1.5 ng mL^−1^	60 min
ELISA Kit for Beta-Lactoglobulin (Biomatik)	ELISA Sandwich, quantitative	β-lactoglobulin	<0.073 ng mL^−1^	4.5 h

## References

[1] Lifschitz C., Szajewska H. (2015). Cow’s milk allergy: Evidence-based diagnosis and management for the practitioner. Eur. J. Pediatr..

[2] Huby R., Dearman R., Kimber I. (2000). Why are some proteins allergens?. Toxicol. Sci..

[3] Council of the European Union Council Regulation No 428/2009 of 5 May 2009 Setting up a Community Regime for the Control of Exports, Transfer, Brokering and Transit of Dual-Use Items (Recast). https://eur-lex.europa.eu/legal-content/EN/TXT/?uri=CELEX%3A32009R0428.

[4] Ylitalo L., Mäkinen-Kiljunen S., Turjanmaa K., Palosuo T., Reunala T. (1999). Cow’s milk casein, a hidden allergen in natural rubber latex gloves. J. Allergy Clin. Immunol..

[5] Courtney R.C., Taylor S.L., Baumert J.L. (2016). Evaluation of Commercial Milk-Specific Lateral Flow Devices. J. Food Prot..

[6] Monaci L., Brohée M., Tregoat V., van Hengel A. (2011). Influence of baking time and matrix effects on the detection of milk allergens in cookie model food system by ELISA. Food Chem..

[7] Monaci L., Visconti A. (2010). Immunochemical and DNA-based methods in food allergen analysis and quality assurance perspectives. Trends Food Sci. Technol..

[8] Schubert-Ullrich P., Rudolf J., Ansari P., Galler B., Führer M., Molinelli A., Baumgartner S. (2009). Commercialized rapid immunoanalytical tests for determination of allergenic food proteins: An overview. Anal. Bioanal. Chem..

[9] Cho C.Y., Nowatzke W., Oliver K., Garber E.A.E. (2015). Multiplex detection of food allergens and gluten. Anal. Bioanal. Chem..

[10] Johnson P.E., Rigby N.M., Dainty J.R., Mackie A.R., Immer U.U., Rogers A., Titchener P., Shoji M., Ryan A., Mata L. (2014). A multi-laboratory evaluation of a clinically-validated incurred quality control material for analysis of allergens in food. Food Chem..

[11] Morgan F., Bouhallab S., Mollé D., Henry G., Maubois J., Léonil J. (1998). Lactolation of β-Lactoglobulin Monitored by Electrospray Ionisation Mass Spectrometry. Int. Dairy J..

[12] Croote D., Quake S.R., Schmidt A., Aebersold R., MacCoss M.J. (2016). Food allergen detection by mass spectrometry: The role of systems biology. NPJ Syst. Biol. Appl..

[13] Ashley J., Shahbazi M., Kant K., Chidambara V.A., Wolff A., Bang D.D., Sun Y. (2017). Molecularly Imprinted Polymers for Sample Preparation and Biosensing in Food analysis: Progress and Perspectives. Biosens. Bioelectron..

[14] Salam F., Uludag Y., Tothill I.E. (2013). Real-time and sensitive detection of Salmonella Typhimurium using an automated quartz crystal microbalance (QCM) instrument with nanoparticles amplification. Talanta.

[15] Tothill I.E. (2011). Biosensors and nanomaterials and their application for mycotoxin determination. World Mycotoxin J..

[16] Yman I.M., Eriksson A., Johansson M.A., Hellenäs K. (2006). Food allergen detection with biosensor immunoassays. J. AOAC Int..

[17] Raz S.R., Liu H., Norde W., Bremer M.G.E.G. (2010). Food allergens profiling with an imaging surface plasmon resonance-based biosensor. Anal. Chem..

[18] Vasilescu A., Nunes G., Hayat A., Latif U., Marty J. (2016). Electrochemical Affinity Biosensors Based on Disposable Screen-Printed Electrodes for Detection of Food Allergens. Sensors.

[19] Inaba I., Kuramitz H., Sugawara K. (2016). Electrochemical Sensing of Casein Based on the Interaction between Its Phosphate Groups and a Ruthenium (III) Complex. Anal. Sci..

[20] Murray B., Deshaires C. (2000). Monitoring Protein Fouling of Metal Surfaces via a Quartz Crystal Microbalance. J. Colloid Interface Sci..

[21] Ito T., Aoki N., Tsuchiya A., Kaneko S., Suzuki K. (2015). Sequential Analysis of β-Lactoglobulin for Allergen Check Using QCM with a Passive Flow System. Chem. Lett..

[22] Billakanti J.M., Fee C.J., Lane F.R., Kash A.S., Fredericks R. (2010). Simultaneous, quantitative detection of five whey proteins in multiple samples by surface plasmon resonance. Int. Dairy J..

[23] Indyk H.E., Filonzi E.L. (2005). Council of the European Determination of lactoferrin in bovine milk, colostrum and infant formulas by optical biosensor analysis. Int. Dairy J..

[24] Muller-Renaud S., Dupont D., Dulieu P. (2005). Development of a biosensor immunoassay for the quantification of αS1-casein in milk. J. Dairy Res..

[25] Ashley J., Piekarska M., Segers C., Trinh L., Rodgers T., Willey R., Tothill I.E. (2017). An SPR based sensor for allergens detection. Biosens. Bioelectron..

[26] Qin B.Y., Bewley M.C., Creamer L.K., Baker H.M., Baker E.N., Jameson G.B. (1998). Structural basis of the Tanford transition of bovine β-lactoglobulin. Biochemistry.

[27] Chun Y.S., Wen L.C., Ming C.Y., Jen P.H., Mao S.J.T. (2005). Epitope mapping of a monoclonal antibody specific to bovine dry milk: Involvement of residues 66-76 of strand D in thermal denatured ß-Lactoglobulin. J. Biol. Chem..

[28] Monaci L., van Hengel A.J. (2008). Development of a method for the quantification of whey allergen traces in mixed-fruit juices based on liquid chromatography with mass spectrometric detection. J. Chromatogr. A.

[29] Morishita N., Akiyama E., Arikawa N., Iida T., Tase K., Hamaji M., Hiraoka S., Shiroyanagi R., Kamijou S., Matsumoto T. (2006). Evaluation of immunochromatographic test kits for food allergens using processed food models, Shokuhin Eiseigaku zasshi. J. Food Hyg. Soc. Jpn..

[30] Kong X., Wang J., Tang Y., Li D., Zhang N., Jiang J., Liu N. (2012). HPLC Analysis of α-lactalbumin and β-lactoglobulin in Bovine Milk with C 4 and C 18 Column. J. Northeast Agric. Univ..

[31] Wu X., Li Y., Liu B., Feng Y., He W., Liu Z., Liu L., Wang Z., Huang H. (2015). Two-Site Antibody Immunoanalytical Detection of Food Allergens by Surface Plasmon Resonance. Food Anal. Methods.

[32] Stephan O., Weisz N., Vieths S., Weiser T., Rabe B., Vatterott W. (2004). Protein quantification, sandwich ELISA, and real-time PCR used to monitor industrial cleaning procedures for contamination with peanut and celery allergens. J. AOAC Int..

